# Community Mobilization to Promote Vaccine Confidence During a Global Public Health Emergency: Insights from Peel Region and Toronto (Ontario, Canada) a Qualitative Study

**DOI:** 10.1177/11786329251381437

**Published:** 2025-10-08

**Authors:** Denessia Blake-Hepburn, Kadidiatou Kadio, Subrana Rahman, Samiya Abdi, M. Hashim Khan, Shaza A. Fadel, Sara Allin, Anushka Ataullahjan, Erica Di Ruggiero

**Affiliations:** 1 Dalla Lana School of Public Health, University of Toronto, ON, Canada; 2 Public Health Ontario, Toronto, ON, Canada; 3 Asthma and Airways Centre (TWH), University Health Network, Toronto, ON, Canada; 4 Institute of Health Policy Management and Evaluation, University of Toronto, ON, Canada; 5 School of Health Studies, Faculty of Health Sciences, Western University, London, ON, Canada

**Keywords:** community engagement, vaccine confidence, community-based task forces, community agencies, public health emergencies, faith, ethno-racial

## Abstract

**Background:**

The Ontario government launched the High Priority Communities Strategy (HPCS) in December 2020, funding community agencies operating in neighborhoods disproportionately affected by COVID-19 in Durham, Peel, Toronto, York, and Ottawa. Community-led task forces and networks also formed with the aim to increase vaccine confidence and uptake among minoritized communities.

**Objectives:**

To explore how community-led task forces, networks and agencies mobilized and engaged faith-based and ethno-racial communities in Peel Region and Toronto to improve vaccine confidence and uptake, including perceived facilitators and barriers.

**Design:**

Multi-method qualitative study.

**Methods:**

Between June 2023 and March 2024, we conducted ten online focus groups with three task forces and six HPCS-funded community agencies, as well as four key-informant interviews with representatives from two task forces and one network. We used thematic analysis to explore respondents’ perceptions and experiences.

**Results:**

Three key themes emerged. First, community-led task forces, the network and agencies used community mobilization strategies, such as tailored outreach, mitigating vaccine access barriers and leveraging trusted community voices, to improve vaccine confidence and uptake. Second, fostering a sense of community was central to their work, enabled through member engagement and power (knowledge and resource) sharing for collective impact. Third, sustaining community-led efforts was a challenge. The volunteer-driven task forces and network lacked the capacity to formally evaluate their activities or long-term infrastructure, and most disbanded post-pandemic. However, community agencies pivoted to preventative and primary care initiatives under HPCS.

**Conclusion:**

Community-led structures contributed to promoting vaccine uptake among ethno-racial and faith-based communities in hotspot areas. Facilitators included the use of trusted messengers and power sharing, while barriers included short-term funding and challenges sustaining efforts over time. Long-term sustainability of these efforts requires continued investment, sustained infrastructure, and strong community partnerships. Lessons from these findings can help strengthen community-led responses to future public health emergencies.

## Introduction

The COVID-19 pandemic introduced unprecedented challenges globally, with minoritized communities bearing a disproportionate burden due to long-standing health and socioeconomic inequities, and other systemic barriers.^1,2^ Efforts to address vaccine hesitancy—rooted in historical injustices in healthcare, deep-seated distrust of government institutions and misinformation—have been particularly challenging among some faith communities and ethno-racial groups.^3-5^ Community engagement is a vital strategy to foster vaccine confidence, disseminating contextually-relevant information, bridging gaps in trust between communities and organizations, and improving access to vaccination for such minoritized populations.^6-8^

We use the term minoritized to emphasize structural issues. Minoritization is a socially constructed concept as opposed to a naturally occurring phenomenon, which shifts focus to the structural causes underlying inequities.^9^ A group being minoritized does not imply a numerical minority, but rather that they are actively socially excluded. It explicitly acknowledges the power differentials that allow for those who are minoritized to also be systemically oppressed, marginalized and othered.^9^

In Ontario, residents in the most ethnically diverse and socio-economically disadvantaged neighborhoods had significantly higher rates of COVID-19 infection and hospitalizations compared to those in the least marginalized areas.^10^ Peel Region and Toronto emerged as some of the hardest-hit areas in Ontario, with high infection rates and low vaccination rates due to access barriers and vaccine hesitancy.^11^ Peel Region and Toronto have ethnoculturally and economically diverse populations, with more than half of their populations belonging to a minoritized group.^12,13^ In response, the Ontario government introduced the High Priority Communities Strategy (HPCS) to allocate funding to community agencies which collaborated with public health units (PHUs), other community agencies, and municipalities to implement targeted interventions across 15 of the province’s most severely impacted neighborhoods.^14^ Six agencies were funded in Peel region and five in Toronto,^15^ each tailoring services to their communities.^14^

Community agencies play a critical role in meeting the unique needs of diverse populations, fostering trust, and advocating for their communities. These agencies are instrumental in public health emergency responses, engaging in initiatives such as health promotion, education, and advocacy.^16^ Addressing the concerns of faith-based, ethno-racial communities required tailored services in response to their specific needs and cultural contexts.^12^

Alongside these agencies, task forces and networks formed early in the pandemic in response to the disproportionate impact of COVID-19 on minoritized communities in Ontario. The task forces in Peel Region and Toronto included healthcare professionals, researchers, and community leaders who were commissioned or voluntarily convened for a specific mandate. Similarly, COVID-19 networks were informal, collaborative efforts focused on improving health outcomes for minoritized communities. While some initiatives were hyper-local, others extended their work across regions.

Community engagement has been widely studied in the context of public health initiatives. Existing frameworks highlight a continuum of engagement, ranging from informing and consulting communities to mobilization, full collaboration and empowerment.^17^ For instance, how community agencies, task forces and networks mobilized their connections and resources during the pandemic to represent their communities effectively and drive meaningful change is worthy of study.^18,19^

This study aims to address how task forces, networks, and community agencies in Peel Region and Toronto engaged and mobilized to improve vaccine confidence and uptake of COVID-19 vaccines, including perceived facilitators and barriers. It explores community-driven responses to prioritize vaccines in hotspot areas, how community is fostered through collective action and the potential sustainability of community-driven initiatives in the face of evolving public health needs.

## Methods

We conducted a multi-method qualitative research study design involving a combination of focus groups and key informant interviews in Peel and Toronto regions in Ontario, Canada.

### Study Area

The study was conducted in Peel and Toronto due to their ethnocultural diversity and disproportionate COVID-19 impact. Peel Public Health was a study collaborator, with community agency recruitment limited to Peel region, though the task forces and network had catchment areas in Peel and Toronto.

### Recruitment Methods

We used purposive sampling to recruit participants from the task forces, network and community agencies active during the pandemic.

### Recruitment of Task Forces/Networks

DBH compiled a list of primary contacts for five task forces and one network operating across Peel region and Toronto and sought input from members of the research team (EDR, SAF, MHK), to help refine the list. In May 2023, the task forces and network of interest (see Table 1) were contacted via email with an information letter and consent forms and follow ups continued through August 2023. All agreed to participate. Initially, we planned focus groups with 8 to 10 participants from each task force and network. However, due to participants’ work commitments, we conducted small group interviews (2-6 people), supplemented by individual interviews.

**Table 1.** Participants and Data Collection Methods.

	Method of data collection	Number of participants
*Community-based task forces and network operating in Peel region and Toronto*		
Black Scientists’ Task Force on Vaccine Equity (BSTF; Toronto-based)	Focus group	N = 6
Canadian Muslim COVID-19 Task Force (CMCTF; Canada-wide)	Focus group 1	N = 4
	Focus group 2	N = 2
Kol Ha COVID Toronto Jewish Community COVID-19 Task Force (Toronto-based)	Interview	N = 1
Latin American COVID Task Force (LACTF)	Interview 1	N = 1
	Interview 2	N = 1
South Asian COVID-19 Task Force (SACTF; Canada-wide)	Focus group	N = 3
South Asian Health Network (SAHN)	Interview	N = 1
*Lead community agencies (HPCS) in Peel region*		
Canadian Mental Health Association (CMHA)—Peel Dufferin Branch	Focus group	N = 2
Dixie Bloor Neighborhood Drop-in Centre (DBNC)	Focus group	N = 5
Indus Community Services	Focus group	N = 4
Punjabi Community Health Services (PCHS)	Focus group	N = 4
Roots Community Services (RootsCS)	Focus group	N = 4
Wellfort Community Health Services	Focus group	N = 3
Total	N = 10 focus groups N = 4 key-informant interviews Total N of participants = 41	

### Recruitment of Community Agencies in Peel Region

In August 2023, SAF approached contacts from three known Peel community agencies, with DBH following up and identifying three additional agencies. Contacts received an information letter and consent forms. Weekly follow-ups continued into January 2024 to maximize participation.

### Data Collection

The team [DBH, KK, EDR, SAF, SA^1^, AA] developed a semi-structured focus group/interview guide (see Supplementary File S1) with input from our knowledge users (KUs) from Peel Public Health, aligning with Integrated Knowledge Translation (IKT) principles.^20^ Our KUs were involved during the early phases of the study and throughout its implementation. They helped refine the research questions and development of the focus group/interview guide to ensure relevance. The guide was then piloted with research team members and revised based on feedback. Between June 2023 and March 2024, we conducted ten focus groups (DBH conducted eight; MYS conducted two) and four interviews (two each by DBH and MYS), totaling 41 participants; 22 from community agencies, 14 of which were community ambassadors (see Table 1).

Sessions were held via Zoom, lasting 50 to 70 minutes. Verbal consent was obtained before audio recording. The semi-structured ten question focus group/interview guide was used to moderate each session. Audio recordings were transcribed by DBH (8), AK (5) and SR (1). Participants received follow-up emails thanking them for participation and inviting them to share additional documentation (eg, vaccine promotion strategies).

### Analytic Approach

Data analysis was inductive, focusing on the meaning of the participants’ discourse rather than on a predetermined theory or conceptual framework.^21^ We used thematic analysis (TA) to explore respondents’ perceptions and experiences: (1) familiarization with the data, (2) systematic coding of the data and development of a codebook, (3) development of an initial theme and consolidation of the codebook, (4) non-rigid coding guided by the codebook, (5) refinement of the codebook and development of a main theme, (6) report writing.

Data familiarization was carried out by two team members (DBH and KK) who examined noisy data from several reads, which were then transferred to NVivo 14 for coding (step 1). DBH and KK developed two preliminary codebooks based on coding four identical transcripts separately (two of the task forces/network and two community agencies). Guided by our research questions, coding was inductive, capturing relevant insights (step 2). Codes were developed using the focus group/interview guide, and were initially descriptive and specific (eg, remove and mitigate barriers that are known to exclude people from accessing services) (step 2). DBH and KK identified the similarities and differences across the two codebooks and discussed and iterated the identified codes with EDR and then with other authors [SAF, SA, AA]. DBH then drafted a consolidated codebook which was thoroughly reviewed by KK to ensure each code was defined (step 3). DBH and KK proceeded to code the remaining ten transcripts focusing on the content of the respondents’ discourse (step 4). Through in-depth iterative review, DBH and KK refined the codebook by reclassifying, re-organizing or merging codes, and developed main themes and sub-themes. Themes were developed by grouping similar codes together to form coherent narratives. For example, codes such as “outreach strategies” and “removing and mitigating barriers” were grouped together based on what they were intended to achieve (ie, mobilizing communities for vaccine prioritization). Reflexive memos were used to synthesize codes and document emerging themes for analysis (step 5). This article reports on findings related to the following main themes: mobilizing communities for vaccine prioritization in hotspot areas; fostering a sense of community; and sustaining community work and relationships (step 6).

### Trustworthiness and Rigor

To ensure study rigor, we applied techniques which align with Lincoln and Guba’s criteria of credibility, transferability, dependability and confirmability.^22^ *Credibility* was enhanced through use of multiple coders who independently coded a subset of transcripts and iteratively developed a codebook through multiple discussions with each other and with the broader research team to validate interpretations. Reflexive memos were used to document analytic decisions and reflections. *Transferability* was ensured by providing contextually relevant information on the study setting and participants (network, task forces, community agencies) involved in the COVID-19 vaccine rollout in Peel Region and Toronto. *Dependability* was achieved by maintaining an audit trail of the decisions related to the focus group/interview guide (informed by the research questions), codebook revisions, team discussions, and theme development. *Confirmability* was strengthened by systematic use of NVivo 14 to organize and trace coding decisions, reflexive memo-writing, and grounding all themes in participant quotations. These techniques contributed to the trustworthiness of the findings.

## Results

We found that the task forces, network and agencies in our sample were actively involved in the COVID-19 response. They implemented community-driven responses which included identifying hotspot areas and missing populations in public health responses, advocating for these populations and responding to localized needs (eg, addressing access barriers). These organizations fostered community through collective action. Members and staff of the task forces, network and community agencies as well as external actors were committed to the work; this ultimately helped to empower individual members of the community to contribute to COVID-19 prevention efforts. Finally, our results revealed that while community played an instrumental effort in COVID-19 response efforts, sustaining community relationships and community work remains an ongoing challenge; this is partly due to funding and human resource constraints as well as differing health priorities for many of these organizations.

### Mobilizing Communities for Vaccine Prioritization in Hotspot Areas

The task forces, network and community agencies in our sample played a pivotal role in implementing community-driven responses to prioritize vaccines in hotspot areas where ethnoracially minoritized populations live. Hotspot areas refer to geographical areas with higher rates of transmission due to structural inequalities.^23^ These efforts consisted of identifying and advocating for missing populations and blind spots in public health responses; identifying and responding to localized needs of these communities and tackling barriers which impede access to vaccines. While advocating for the equitable distribution of vaccines yielded positive results (eg, building trust amongst community members), there were also challenges, such as pushback from some community groups on vaccine prioritization.

### Identifying Missing Populations and Blind Spots in Population Approaches

The task forces, network and community agencies reported that certain populations were being disproportionately impacted by COVID-19 and some populations (eg, individuals without a health card) were being missed in general population approaches. They recognized that these populations needed to be prioritized in vaccination efforts, while also recognizing existing gaps in the public health response. For example, the task forces and network identified issues with translation of public health orders in different languages and the provision of culturally appropriate information for ethnoracially minoritized populations, which resulted in either no one showing up to vaccine clinics or frustration due to huge lineups. While respondents unanimously reported that their activities were inclusive and equitable, one respondent noted the importance of recognizing blind spots (eg, “who is being left out”), and reflected that the network missed several groups in the work they were doing:

I think equity and inclusivity was the forefront of what we were doing. That’s the reason why we came to be. But at the same time, it’s also very hard to look at the whole spectrum of inclusivity [. . .] We don’t even know who is being left out because we may not necessarily know who they are and how they want to be reached or how they can be reached. We only know who we’re targeting and why we’re targeting. (Interview 4)

### Identifying Localized Needs and Barriers

The task forces, network and community agencies identified barriers that were known to exclude people from services and create inequity. These barriers included linguistic, geographical and inequitable distribution of vaccines and health service payments which impact non-English speakers, newcomers, non-insured individuals and hard-to-reach (eg, incarcerated) populations. These payments specifically impact those who are not covered under the Ontario Health Insurance Plan (OHIP), which provides free or low-cost health care to Ontario residents.^24^

Task force and network respondents shared that they identified the needs and barriers of the communities by using existing communication channels (eg, WhatsApp), engaging with other community actors doing outreach work, and gathering feedback directly from community members through support or help lines, questionnaires and townhall sessions where community members could ask questions. These methods helped to improve outreach approaches and advocate for the community based on their actual needs (eg, provision of wraparound supports such as transportation):

We do have a phone line where clients can call in and share any sort of concerns they have. Sometimes they just call in to ask, “Can you help me find a family doctor near my home?” We have translation services for that phone line. And we do strive to provide various mediums [in person, email] for people to connect with us. . .because sometimes people don’t have access to internet. (Focus group 5)

The task forces, network and agencies provided COVID-19 vaccine-related information (through training sessions, workshops, videos, infographics, social media and communication channels) to their respective communities based on their identified religious and language needs.

### Responses to Address Localized Needs and Barriers

The task forces and network were proactive in identifying priorities, and bridging gaps in public health responses (eg, delayed recommendations about COVID-19 transmission, translation of public health orders into different languages). Many of the task forces and network drew on local and international data about how COVID-19 was spreading from other countries (eg, Spain, UK, USA) to inform decision making and to take preventative action. For instance, some of the task forces and network took a proactive approach to get ahead of the pervasive and harmful public narrative that South Asian communities were to be blamed for the spreading of COVID-19 in Peel due to gatherings. They provided evidence that indicated that drivers of increased prevalence included essential and precarious work, lack of vaccine access, and multigenerational homes. These task forces and network released guidelines to help their communities stay safe during major holidays and gatherings, which were established as community transmission risk factors.

The task forces, network and community agencies sought to remove and mitigate the barriers (eg, linguistic, geographical) that were known to exclude people from accessing services. They reported using a variety of means to improve equitable access, such as vaccine clinics with no OHIP requirement or mobile clinics in geographical areas with low vaccination rates; and holding townhalls accessible through social media and ethnic media (ie, media produced by and for ethnic communities) platforms. These organizations developed culturally relevant and sensitive material, including translation of public health information in multiple languages for poster campaigns, infographics, guidelines and websites. They also provided additional referrals of services and wrap-around supports:

One of the things that this program helped to do was also mitigate the effects of people waiting to receive support because we are able to provide that initial support. Along with the lens of wraparound supports as well. . .we’re able to ensure that we’re able to connect them with any kind of financial help, food security, childcare supports, social supports and settlement supports [. . .] the client is able to call through our support line and then be connected to various different resources. . .to make sure that they receive the services that they need. (Focus group 10)

### Advocating for Equitable Distribution of vaccines

Respondents shared challenges in advocating for the equitable distribution of vaccines. Respondents from task forces expressed they did not know who to contact (eg, local government representatives, public health leaders) to inform public health decision-making. One respondent reflected that much of the advocacy that led to vaccine prioritization in hot spot areas was done by community groups (eg, task forces and network). However, advocacy was at times met with pushback from people (eg, decision-makers) who had competing views on which groups should be prioritized (eg, seniors in long-term care homes versus seniors living in other community settings). Continued advocacy did, however, lead to prioritization by hotspot areas (eg, South Asian seniors living in high-risk community settings) by the Ontario government.

On the other hand, one task force revealed that advocating for communities who were disproportionately impacted by COVID-19 led to the task force being viewed as a trusted source of information. Part of this advocacy included calling for the collection of race-based data to ensure better representation of racialized communities in the data and investment in racialized communities to prepare for the next pandemic:

I think community trusted us because we were advocating for them to different levels of government. We had representations around needing race-based data so that we could actually have accurate information in terms of our community and other communities taking the vaccine. . .We were lobbying in terms of ensuring that there were popup clinics in areas where the Black community was prominent. (Focus group 1)

### Fostering a Sense of Community Through Collective Action

The work of the task forces, network and community agencies helped to foster community in several ways. Their work fostered community between members involved in this work; between community actors (ie, ambassadors, task force and network members) and community members; and ultimately between individuals within communities. The process of fostering community began with strong volunteer commitment from community actors, followed by engagement with different actors which facilitated power sharing. This process contributed to empowering individuals within communities to act to take the COVID-19 vaccine.

### Strong Volunteer Commitment from Actors

Task force and network members were committed to the voluntary work. Members took on leadership positions because they were coming from the communities that COVID-19 was devastating; and understood that community had to come together to address the disproportionate impacts of COVID-19 on their communities. A volunteering ethos was one of several ways in which community was fostered:

I think the first month of the task force, our meetings were 9:30 PM which was the time people, after they come back or finish their routines after dinner, after family time. Or it was 6 in the morning, because then by 7, the doctors were jumping on calls already for meetings. And. . .it took a lot of organizing, but. . .it was a strong commitment from the providers to be part of this and to continue to translate. (Interview 1)

### Community Engagement and Power (Knowledge and Resource) Sharing for Collective Impact

Engagement with different actors was instrumental to building community and collective community work; it facilitated power (both knowledge and resource) sharing. For example, the task forces, network and community agencies leveraged existing community relationships and ties through organizations to reach different populations with which they did not have existing access. Community agencies engaged with other agencies on an ongoing basis through meetings, to share and exchange information about strategies (eg, outreach locations) and challenges. Each agency was assigned their own catchment areas; however, the work was not limited to these areas. The agencies collaborated and supported (ie, translation of material, support at clinics, townhalls) each other where necessary. Collaboration made it easier for community agencies to gain access to different communities to provide supports. For instance, community agencies sometimes partnered with other agencies, under the HPCS, to access specific populations:

Regarding the Black community, we partnered with Roots Community Services and we did a joint outreach in the buildings where majority of people are from Black communities. We work together and did health promotion for [COVID-19] vaccine. (Focus group 7)

The task forces, network and other community groups mobilized together to share knowledge and resources, including to implement mass awareness campaigns (eg, “This Is Our Shot”) to address vaccine confidence among the populations that were being disproportionately affected by COVID-19. For some task forces and network, shared cultural backgrounds led to natural collaboration. The task forces and network also reported that they shared their resources (eg, Zoom license) with other task forces, community groups and organizations to organize outreach activities (eg, town halls). Knowledge and resource-sharing helped task forces replicate the structure and work of other task forces. The task forces and network also reported engaging with community agencies operating in Peel to identify the needs of communities and maintained regular communication.

### Community Work Empowers Other Community Members Through Representation

Many task forces and network reflected that they had formed to empower their community members to take preventative action during the pandemic. Community representation was at the crux of empowering community members. Task force and network members, and ambassadors working for community agencies looked like and spoke the same languages as the communities who were the intended audience of the outreach work. Representation helped to bridge trust and communication with their respective communities and empowered community members to act.

The task forces and network shared that they implemented culturally relevant approaches (eg, cultural music, foods) to their work (eg, vaccine clinics, town halls) to empower community members to act, given that they themselves had the cultural insight. For instance, townhalls included panelists who looked like and spoke the same languages as their audiences which helped to foster community, “dismantle mistrust” and promote COVID-19 vaccines. Ambassadors from community agencies convinced community members to attend vaccine clinics by emphasizing that the healthcare professionals who would be administering vaccines were representative of these community members.

Respondents reflected on how power comes with representation (being from the same community) and how that relates to building trust amongst community members:

I feel so much power and representation. Seeing yourself, someone who looks like you, someone who speaks the same language as you, someone who understands what your concerns are, makes a huge difference in people [. . .] It’s just they need that connection with someone who understands them because they are like them. And I think that in and of itself builds so much trust within our community, just the fact that we were from the same community. (Interview 4)

Ambassadors, paid through community agencies, were often also members of the community. The creation of ambassadors or “public champions” helped to foster community by empowering fellow community members to take on the responsibility to act (getting vaccinated and encouraging others to get vaccinated). Ambassadors took a proactive approach to “champion themselves” to take the vaccine and promote it amongst their community members. Some ambassadors found that by taking the vaccine, others felt comfortable following suit.

### Sustaining Community Relationships and Community Work Remains an Ongoing Challenge

While COVID-19 vaccination is no longer a pressing focus for these task forces, network and community agencies, sustainability of community relationships and work remains an ongoing challenge. Differences for the task forces and network compared to community agencies were observed. Respondents reflected on the facilitating and hindering factors that may impact the ongoing sustainability of their community work (eg, outreach) in the face of changing pandemic needs, such as funding levels and sources. Respondents from the task forces and network shared their perspectives on ways in which community voices can be amplified through means such as inclusion of these voices in decision-making at the institutional level and increased funding.

#### Building Community Is Not Without Its Challenges

The process of building community was not always linear or easy. Respondents revealed several challenges engaging with different actors (eg, health system, community-based organizations), including the lack of support from some organizations (eg, public health, faith-based organizations).

One respondent shared that their task force received recognition and a motion by health system actors to continue as a task force. Task force members considered their work a success given that health system actors (eg, Board of Health) viewed their work as a model for addressing other health issues (eg, cancer screening). While respondents reported receiving support from public health, one respondent shared that their task force received more support from one PHU versus another.

I didn’t feel that much engagement from [XXX] Public Health, but again it might have been asked on us to reach out more maybe, but we tried to reach a few times and we weren’t successful, but [XXX] did. (Interview 1)

Another respondent reflected that PHUs did not initially see their volunteer driven task force/network as a credible resource until they were able to demonstrate and see the value of their work; this perception was contrasted with other volunteer-driven community organizations which were helpful and immediately saw the “value of their work”. Similarly, some faith-based organizations (eg, churches, mosques) were supportive and some were not supportive or resistant of the task forces, network and community agencies coming into their space to do vaccine promotion. Some of these organizations were resistant due to religious beliefs (“God will protect us”) or because they were weary of targeted vaccine promotion (“*Why don’t you guys cater to everyone? Why do you guys focus primarily on the BAC [Black, African, Caribbean] community?*”).

#### Funding Levels and Sources

Community agencies received funding from Ontario Health for the HPCS project with a focus being on vulnerable communities, while the task forces and network were not formal organizations and were volunteer driven. Many of the task forces and network received very limited to no funding and therefore had to organize the structure and activities around available financial resources or lack thereof. The task forces and network mobilized their own financial (paying out-of-pocket) resources to implement outreach activities:

And for one of our largest clinics in Toronto, one of the first one we actually did collect money among us to buy t-shirts. And we had an artist. . .And at that time, it wasn’t a huge thing, but I think we chose something like $50 each and we got the t-shirts. (Interview 1)

Provision of funding from external organizations gave task forces and network the resources which helped them do the work. For instance, one PHU connected one organization with a funding opportunity through an organization called CASSA [The Council of Agencies Serving South Asians]. One respondent expressed the frustration in organizations that were successful in securing federal funding to create materials for racialized communities but had no apparent connection to the communities. The respondent reflected that more emphasis should be placed on supporting (eg, funding) and amplifying the efforts of communities who are already doing the work, rather than funding organizations who have no connection to those communities.

#### Impact of Funding on Evaluation

Given the lack of funding and financial resources, the task forces and network did not have the capacity to formally evaluate their work. Rather, the groups relied on informal evaluation methods such as: collection of quantitative data (eg, number of vaccines administered, numbers of volunteers, number of clinics supported); pre and post-tests (eg, townhalls); community feedback; and tracking levels of vaccine hesitancy and uptake via social media. The task forces and network also looked at indirect data collected by public health on strategies and what was successful. In comparison, community agencies were government-funded and had the resources to evaluate their work. Public health and community agencies collected data and key performance indicators (eg, the number of vaccines delivered by forward sortation areas (FSAs)) as required by Ontario Health.

While task forces/network expressed not being able to sustain or properly evaluate their work because of lack of funding and not being seen as a formal organization, one respondent expressed that not being seen as a registered charity and the lack of funding made members more innovative, creative and fostered relationship building. Multiple people working helped to mitigate the challenge of funding and commitment:

I think when you are a fully-fledged organization, you have to work through the mandates of the organization, you’re constrained with what the organization receives funding for, who is getting paid for what job, etc. . .because we were just all volunteers it was easy to do essentially whatever was required of us. And we didn’t have any of those constraints, other than obviously time constraints. . .Funding was a huge challenge. But it made us more creative and innovative in how we did things. And maybe also helped us build relationships, because we’re like, “we’ve got no money, but we’re not in it for the money either” . . .It wasn’t transactional at that point. It was beyond transactional. And I thought that was the beauty of it all. (Interview 4)

#### Status and Shift in Focus of Organization

Many of the task forces and network have disbanded completely or continue to operate on a smaller scale and have shifted their focus to providing medical advice and/or addressing other health issues (eg, mental health, diabetes and cardiovascular) impacting their respective communities. Respondents cited that the work decreased, or they disbanded due to COVID-19 burnout and COVID-19 being less of a public emergency. As for community agencies, COVID-19 work has decreased, and their work has shifted to post-pandemic and prevention-based care (eg, cancer screening, diabetes, mental health):

We’ve shifted from the COVID-19 lens. Not that it’s not important. We still do talk about it here and there, but it’s not our main focus as it has passed. We are still high priority for the vulnerable communities because there’s a lot more that needs to be targeted, like mental health for example, access to primary physicians, getting your checkups and all of that. (Focus group 8)

#### Leveraging the Value of Insider Community

The task forces and network were formed by community, some of whom were also health professionals with insider knowledge. Respondents reflected that health system actors should look to work with communities and leverage this insider knowledge in the future. One task force reflected on the importance of hard-hit communities having voices at the table at institutional levels, who can inform decision-making in preparation for the next pandemic:

It would be really important for “hard-hit” [interpreted from ‘hardy’] communities like Brampton to have a voice at the table. Our task force, we tried so much. . .We couldn’t even get to the table. We had a couple meetings with Dr. Bogoch and that was it. But I think we really need to make sure that physician led grassroots organizations like the task force who understand the community, have trust in the community, be at the table at the institutional level and do that now. (Focus group 2)

## Discussion

While there is growing literature on the mechanisms in which health systems and governments can facilitate community engagement as a response to public health emergencies,^25^ our study directly explores community engagement experiences in pandemic planning and responses from the perspectives of community mobilizers. This study fills a gap on the processes of community engagement at a meso-level (task forces, networks and agencies) and explores how these community structures operated in Peel Region and Toronto during the COVID-19 pandemic. This analysis complements the findings reported in another paper which explores how the task forces, network and community agencies facilitated trust at interpersonal and organizational levels among their community members in Peel Region and Toronto during the COVID-19 pandemic.^26^

The task forces, network and community agencies implemented community-driven responses to prioritize vaccines in hotspot areas where ethnoracially minoritized populations lived. Responses involved identifying and responding to the needs of the communities and the access barriers these communities face. The work of these community structures helped to foster a sense of community between members (eg, task force members) involved in this work; between the community actors (eg, ambassadors) and community members; and ultimately between community members. These qualitative insights are further supported by vaccination uptake data. In Peel Region, community-based clinics reached underserved neighborhoods with low vaccine coverage—delivering nearly 100 000 doses and driving an 11% increase in first dose coverage, reinforcing the value of localized strategies and community engagement frameworks.^27^

While vaccination is no longer a pressing focus, we found that the sustainability of their respective work differs for the task forces and network compared to community agencies. Facilitating and hindering factors that may impact the ongoing sustainability of their community work (eg, outreach) included funding levels and sources. Respondents from the task forces and network call for community voices to be amplified through the inclusion of these voices in decision-making at the institutional level, increased funding, and the collection of race-based data.

Our findings emphasize the importance of planning for sustainability at the onset of community engagement efforts. It is necessary to initiate relationship building early with communities who have insider knowledge to foster their sense of empowerment and ownership.^25^ Structural supports are also necessary^28^ to aid communities in mobilizing their assets and providing necessary resources to develop community capacities (ie, skills, competencies).^25^ Cross-sectoral partnerships between different actors (eg, municipal, health system, community) may help diversify resources, however care must be taken to not shift responsibility away from government actors who are critical to addressing systemic inequalities.^29^ Further, our findings have shown that the experience of community groups working with different actors like public health units was not monolithic, rather the level of engagement varied, emphasizing the importance of a consistently applied framework for partnership.

As our findings suggest, meaningful community engagement requires power sharing in terms of knowledge and resources, working closely with communities to understand their needs and preferences for levels of engagement in efforts.^28,30^ Engaging with health centers, faith-based and other community agencies and local physicians can help health system actors identify trusted messengers, improve and incorporate community knowledge and advance public health priorities.^31^ Health system actors should better engage underrepresented community voices in the development, implementation and evaluation of healthcare programs and policies.^28^ However, more harm can be done if these actors do not first acknowledge the inequities and historical and continued atrocities faced by minoritized communities.^32^

Therefore, health system actors should carefully consider the use of language when engaging with these communities. “Stigmatizing and discriminatory language perpetuates and increases harm to people and communities who have been marginalized by systems of oppression”.^33^ To meaningfully engage in equity work requires using terms that counter rather than reinforce power imbalances, moving beyond language that focuses on the individual and instead draws attention to the multifaceted root causes of injustice; for example, “marginalization created by systems of oppression” versus “marginalized groups”.^33^

Many of the task forces and network reported that the ethical collection of race-based data is necessary to ensure there is better representation of minoritized communities in data and public health emergency responses. While public health officials are well positioned to collect this data,^34^ PHUs reported capacity (ie, organizational, operational) issues.^35^ As shown in our results, with the absence of race-based data, decision-makers still gained valuable insights and lessons from community feedback and other jurisdictions which emphasized the disproportionate impact on ethnoracially minoritized populations.^34,36^ Better provincial infrastructure is necessary to routinely collect race-based data to identify at-risk communities, address access barriers,^35^ and drivers of health inequities^37^; this includes providing adequate resources to PHUs and directly involving ethnoracially minoritized communities in the ethical collection, analysis and use of race-based data.^37^

### Limitations

Our study is not without limitations. We experienced challenges with recruitment and participation for a variety of reasons including, most of the task forces and the network disbanding and health professionals returning to their full-time jobs, difficulty getting people’s schedules aligned or reaching the appropriate contacts. Therefore, some focus groups were not as representative of their organizations or task force and network. Another limitation (due to feasibility) is that our data only captured the perspectives of community leaders (ie, task force and network members, staff from community agencies) which may not comprehensively reflect the opinions of individuals from their respective communities. Interrogating the different “meanings” of community was also beyond the scope of our study. However, our study still contributes a substantial perspective as members of these task forces and network, and staff from community agencies were representative of the communities they serve.

## Conclusion

This study explored how these 12 community-led task forces, network and agencies mobilized and engaged faith-based and ethno-racial communities to improve vaccine confidence and uptake of COVID-19 vaccines in Peel Region and Toronto. Study findings highlight how community actors contributed to delivering tailored, trust-based outreach, mitigating vaccine access barriers and fostering collective engagement to improve vaccine uptake. Key facilitators included the use of trusted messengers and power sharing, while barriers included short-term funding and challenges sustaining efforts over time. These findings further support the need for a more nuanced examination of community engagement processes. These findings may also help to strengthen community-led responses to public health emergencies in similar local contexts. Future research should examine potential community mobilization strategies for long-term sustainability.

## Supplemental Material

sj-docx-1-his-10.1177_11786329251381437 – Supplemental material for Community Mobilization to Promote Vaccine Confidence During a Global Public Health Emergency: Insights fromPeel Region and Toronto (Ontario, Canada) a Qualitative Study Supplemental material, sj-docx-1-his-10.1177_11786329251381437 for Community Mobilization to Promote Vaccine Confidence During a Global Public Health Emergency: Insights fromPeel Region and Toronto (Ontario, Canada) a Qualitative Study by Denessia Blake-Hepburn, Kadidiatou Kadio, Subrana Rahman, Samiya Abdi, M. Hashim Khan, Shaza A. Fadel, Sara Allin, Anushka Ataullahjan and Erica Di Ruggiero in Health Services Insights
