# Supplementary material for: Community Mobilization to Promote Vaccine Confidence During a Global Public Health Emergency: Insights from Peel Region and Toronto (Ontario, Canada) a Qualitative Study
Source: Health Serv Insights. 2025 Oct 8;18:11786329251381437. doi: 10.1177/11786329251381437 (PMC12515287; doi:10.1177/11786329251381437)
Supplement: sj-docx-1-his-10.1177_11786329251381437 – Supplemental material for Community Mobilization to Promote Vaccine Confidence During a Global Public Health Emergency: Insights fromPeel Region and Toronto (Ontario, Canada) a Qualitative Study [file sj-docx-1-his-10.1177_11786329251381437.docx]

**Focus group/ Interview Guide for Task Forces/Networks/Community Agencies working with Faith, Racial and Ethnic Communities**

**Sample questions from the focus group/interview guide:**

1. *Please tell us about your role in the Task Force/Network/Community Agency and how long have you been involved.*
2. When was your Task Force/Network/Community Agency established? (Probe for its origins, reasons, motivations, initiators)
3. *Now we would like to discuss your Task Force/Network/Community Agency’s activities*:
4. Please take a moment to recall a collaboration between your Task Force/Network/Community Agency and other organizations (e.g. public health unit, other faith-based groups) to promote vaccine uptake (e.g., vaccine drives, education sessions, Q&As, et cetera). What meaningful activities did the Task Force/Network/Community Agency perform?
5. Was your Task Force/Network/Community Agency able to promote trust in vaccines?
6. How did you measure the success/impact of your activities? Can you give examples?
7. *We have reached the end of our focus group/interview:*
8. Would you be able to share any relevant documentation with us after this focus group/interview?
